# Selecting statistical model and optimum maintenance policy: a case study of hydraulic pump

**DOI:** 10.1186/s40064-016-2619-1

**Published:** 2016-07-04

**Authors:** S. Ruhi, M. R. Karim

**Affiliations:** Department of Statistics, Pabna University of Science and Technology, Pabna, Bangladesh; Department of Statistics, University of Rajshahi, Rajshahi, 6205 Bangladesh

**Keywords:** Case study, EM algorithm, Hydraulic pumps, Maintenance data, Mixture models, Reliability

## Abstract

**Introduction:**

Proper maintenance policy can play a vital role for effective investigation of product reliability. Every engineered object such as product, plant or infrastructure needs preventive and corrective maintenance.

**Case description:**

In this paper we look at a real case study. It deals with the maintenance of hydraulic pumps used in excavators by a mining company. We obtain the data that the owner had collected and carry out an analysis and building models for pump failures. The data consist of both failure and censored lifetimes of the hydraulic pump.

**Discussion and evaluation:**

Different competitive mixture models are applied to analyze a set of maintenance data of a hydraulic pump. Various characteristics of the mixture models, such as the cumulative distribution function, reliability function, mean time to failure, etc. are estimated to assess the reliability of the pump. Akaike Information Criterion, adjusted Anderson–Darling test statistic, Kolmogrov–Smirnov test statistic and root mean square error are considered to select the suitable models among a set of competitive models. The maximum likelihood estimation method via the EM algorithm is applied mainly for estimating the parameters of the models and reliability related quantities.

**Conclusions:**

In this study, it is found that a threefold mixture model (Weibull–Normal–Exponential) fits well for the hydraulic pump failures data set. This paper also illustrates how a suitable statistical model can be applied to estimate the optimum maintenance period at a minimum cost of a hydraulic pump.

## Introduction

Every engineered object (product, plant or infrastructure) needs preventive and corrective maintenance. The cost of maintenance can vary from 5 to 30 % (Campbell 1995) of the operating budget depending on the industry sector. This implies that businesses need to manage maintenance effectively to ensure minimum costs. This requires proper data management to assist in building models for effective decision making.

In this paper we look at a real case study. It deals with the maintenance of hydraulic pumps used in excavators by a mining company. We look at the data that the owner (mining company) had collected and carry out an analysis and build models for pump failures. The data given in Murthy et al. (2015) and Karim et al. (2015) consist of both failure and censored lifetimes of the pump. Murthy et al. (2015) and Karim et al. (2015) showed that the threefold Weibull mixture distribution is the best distribution for the data among the three competing distributions (single Weibull, twofold Weibull mixture and threefold Weibull mixture). In this paper we search a suitable distribution for the data from a set of competitive mixture models (based on Weibull, Exponential, Normal and Lognormal distributions). Finally the selected distribution is used to find out the optimum time at which the expected cost for maintenance of the pump will be minimum.

The remainder of the article is organized as follows: “Hydraulic pump failure data” section describes a set of hydraulic pump failure data which will be analyzed in this paper. “Mixture models for modeling failure data” section presents the mixture models for modeling failure data. “Parameter estimation” section presents the MLEs of the parameters of mixture models by applying the Expectation–Maximization (EM) algorithm. “Model selection” section describes about the model selection for the data through graphical and statistical approaches. “Optimum maintenance cost” section expresses a procedure in which we have tried to find out the optimum time at which the expected cost for maintenance of the pump will be minimum. Finally, “Conclusion” section concludes the article with a discussion of the key findings.

## Hydraulic pump failure data

The hydraulic pumps considered here are used in excavators by a mining company. In open cut mines, coal and overburden are transported using excavators and dump trucks. An excavator is a complex machine consisting of several systems. The hydraulic system is one of the important systems comprised of several hydraulic pumps (for linear and rotational motions), hydraulic oil filters and several hydraulic lines. A pump is considered to have failed if it cannot provide the required flow rate at the required pressure. The data recorded by the maintenance department consist of the failure times (for units that have failed and required Corrective Maintenance action) and service times (for units that have not failed yet and were sent for Preventive Maintenance action) for 102 U and presented in Table 1. The column, labeled “Age” means the age (in hours) of the item at the end of the data collection period and the column labeled “Type” indicates whether the data is a failure data (denoted by 1) or censored data (denoted by 0). As can be seen the data consists of 45 failures and 57 censored ages. More detail description of the data can be found in Murthy et al. (2015) and Karim et al. (2015).

**Table 1:** Hydraulic pump failure data

Age (h)	Type	Age (h)	Type	Age (h)	Type	Age (h)	Type
81	0	3333	1	9334	1	12,198	0
149	1	3569	1	9368	1	12,198	0
245	1	3837	0	9729	1	12,198	0
340	1	3837	0	9751	0	12,198	0
407	1	4150	0	10,299	1	12,236	0
461	1	5123	1	10,389	0	12,236	0
629	1	5258	1	10,413	0	12,236	0
856	0	5662	0	10,557	1	12,236	0
947	0	5923	1	10,944	1	12,236	0
1460	1	6333	1	10,970	1	12,236	0
1513	1	6717	1	11,647	0	12,394	0
1670	1	7207	1	11,678	1	12,459	0
1688	0	7265	1	11,686	1	13,097	0
2093	0	7624	1	11,798	0	13,497	0
2242	0	7625	0	11,869	0	13,497	0
2242	0	7973	1	11,869	0	13,497	0
2242	0	8183	1	11,923	0	13,497	0
2242	0	8217	1	12,005	0	13,497	0
2242	0	8390	1	12,082	0	13,497	0
2607	1	8462	1	12,090	0	13,497	0
2668	1	8728	1	12,136	0	14,407	1
2806	1	8817	1	12,141	0	15,536	1
3132	0	8870	1	12,143	0	16,289	1
3132	0	8884	0	12,163	0	17,517	1
3132	0	9055	1	12,198	0		
3132	0	9182	1	12,198	0		

## Mixture models for modeling failure data

A variety of statistical models have been developed and studied extensively in the analysis of product failure data (Kalbfleisch and Prentice 1980; Meeker and Escobar 1998; Blischke and Murthy 2000; Lawless 2003; Murthy et al. 2004). A set of mixture models that have been used to analyze the pump failure data, given in Table 1, are discussed below.

The cumulative distribution function (cdf) of a general *n*-fold mixture model involves *n* subpopulations is given by

1 $$G(t) = \sum\limits_{i = 1}^{n} {p_{i} F_{i} \left( t \right)} ,\quad t \ge 0$$

where $p_{i} > 0$ and $\sum\nolimits_{i = 1}^{n} {p_{i} = 1}$. Here $F_{i} \left( t \right)$ is the cdf of the *i*-th sub-population and $p_{i}$ is the mixing probability of the *i*-th sub-population. The corresponding probability density function (pdf) is given by

2 $$g(t) = \sum\limits_{i = 1}^{n} {p_{i} f_{i} (t)} ,\quad t \ge 0$$

where $f_{i} (t)$ is the pdf associated with *F*_*i*_(*t*). And the reliability function is

3 $$R\left( t \right) = 1 - G\left( t \right) = 1 - \sum\limits_{i = 1}^{n} {p_{i} F_{i} \left( t \right)} \,,\quad t \ge 0.$$

The cumulative distribution functions, probability density functions and reliability functions for the various twofold and threefold mixture models can be obtained from Eqs. (1)–(3) by putting *n* = 2 and *n* = 3, respectively. Ruhi et al. (2015) applied a twofold Weibull mixture model for analyzing failure data. More literatures on the applications of mixture models can be found in Titterington et al. (1985), Mendenhall and Hader (1958), Ahmad and Abdelrahman (1994), and Murthy et al. (2004).

## Parameter estimation

We estimate the parameters of different mixture models by applying the maximum likelihood estimation method. We apply the Expectation–Maximization (EM) algorithm to find the maximum likelihood estimates (MLEs) of the parameters. Details on the application of EM algorithm for mixture models with censored data can be found in Ateya (2012), Bordes and Chauveau (2012) and Ruhi, et al. (2015). Karim, et al. (2015) have applied single Weibull, twofold Weibull mixture and threefold Weibull mixture models for this data set and suggested the threefold Weibull mixture model as the best fitted model on the basis of various graphical and statistical approaches. In addition to threefold Weibull mixture model, here we have assumed two other threefold mixture models (Weibull-Normal-Exponential and Normal-Lognormal-Weibull) for the data. Our aim is to find out whether any other threefold mixture model fits this data set better than the threefold Weibull mixture model or not. And if the distribution changed, what would be its effect on optimal maintenance policy.

The parameters of these three mixture models are estimated by applying maximum likelihood method via the Expectation–Maximization (EM) algorithm. R programming codes are written for all computations of the paper. Programming codes for analyzing the data with Weibull–Normal–Exponential mixture model are given in the “Appendix”. The given codes can be used for other two models after simple modifications, mainly related to the functions dweibull(), pweibull(), dnorm(), pnorm(), dexp() and pexp() and the parameter vector theta.

The MLEs of the parameters are displayed in Table 2. In Table 2, the parameters, *p*_1_, *p*_2_, and *p*_3_ represent the mixing probabilities of the 1st, 2nd and 3rd sub-populations, respectively.

**Table 2:** MLEs of the parameters of assumed models

Threefold mixture models	MLEs of parameters
Weibull (β_1_, η_1_)–Weibull (β_2_, η_2_)–Weibull (β_3_, η_3_)	$$\begin{aligned} & \left\{ {\beta_{1} ,\eta_{1} ,\beta_{2} ,\eta_{2} ,\beta_{3} ,\eta_{3} ,p_{1} ,p_{2} ,p_{3} } \right\} = \\ & \{ 1.0191,\;2364.0191,\;5.5758,\;9481.8351,\; \\ & 16.6426,\;16535.5039, \, 0.1659,\;0.3220,\;0.5120\} \\ \end{aligned}$$
Weibull (β, η)–Normal (μ, σ)–Exponential (δ)	$$\begin{aligned} & \left\{ {\beta ,\eta ,\mu ,\sigma ,\delta ,p_{1} ,p_{2} ,p_{3} } \right\} = \\ & \{ 5.5391,\;9527.83,\;15991.11,\;1073.821,\; \\ & 0.0004,\;0.3249,\;0.5076,\;0.1674\} \\ \end{aligned}$$
Normal (μ_1_, σ_1_)–Lognormal (μ_2_, σ_2_)–Weibull (β, η)	$$\begin{aligned} & \left\{ {\mu_{1} ,\sigma_{1} ,\mu_{2} ,\sigma_{2} ,\beta ,\eta ,p_{1} ,p_{2} ,p_{3} } \right\} = \\ & \{ 15992.0308,\;1072.7513,\;7.5063,\;1.3759,\; \\ & 5.4782,\;9497.0899,\;0.4947,\;0.1872,\;0.3180\} \\ \end{aligned}$$

### Comment

- For Weibull (β_1_, η_1_)–Weibull (β_2_, η_2_)–Weibull (β_3_, η_3_) mixture model, the mean for $F_{3} \left( {t;\beta_{3} ,\eta_{3} } \right)$ = 16,018.005 > mean for $F_{2} \left( {t;\beta_{2} ,\eta_{2} } \right)$ = 8760.457 > mean for $F_{1} \left( {t;\beta_{1} ,\eta_{1} } \right)$ = 2345.628.
- For Weibull (β, η)–Normal (μ, σ)–Exponential (δ) mixture model, the mean for $F_{2} \left( {t;\mu ,\sigma } \right)$ = 15,991.110 > mean for $F_{1} \left( {t;\beta ,\eta } \right)$ = 8799.642 > mean for $F_{3} \left( {t;\delta } \right)$ = 2500.000.
- For Normal (μ_1_, σ_1_)–Lognormal (μ_2_, σ_2_)–Weibull (β, η) mixture model, the mean for $F_{1} \left( {t;\mu_{1} ,\sigma_{1} } \right)$ = 15,992.031 > mean for $F_{3} \left( {t;\beta ,\eta } \right)$ = 8765.749 > mean for $F_{2} \left( {t;\mu_{2} ,\sigma_{2} } \right)$ = 4688.418.

## Model selection

**Fig. 1:**
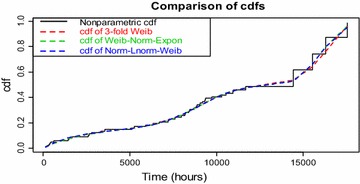
Comparison of parametric and nonparametric estimates of cdfs

This section applies the graphical and statistical approaches for selecting the best fitted model for the data set among three competitive threefold mixture models listed in Table 2. A relatively straightforward approach to select a tentative model is to utilize the plotting methodology where the cdfs obtained from parametric estimates are compared with the empirical distribution function. More detail about this comparison can be found in Blischke et al. (2011). The cdfs of threefold Weibull, Weibull–Normal–Exponential and Normal–Lognormal–Weibull mixture models are compared with the empirical distribution function (nonparametric estimate of cdf from Kaplan–Meier (KM) estimate) and the results are displayed in Fig. 1.

Figure 1 indicates that all the cdfs obtained from the three different mixture models give approximately same result, except at the right tail of the figure of cdfs, where the cdfs of Weibull–Normal–Exponential and Normal–Lognormal–Weibull mixture models belong slightly closer to the nonparametric estimate of cdf than that of the cdf of threefold Weibull mixture model. Hence we may consider both the Weibull–Normal–Exponential and Normal–Lognormal–Weibull mixture models for the data set.

The statistical approaches provide a more rigorous method for model selection and validation. Various statistics [such as adjusted Anderson–Darling (AD*) value, Kolmogrov–Smirnov (KS) test statistic, Akaike Information Criterion (AIC) and root mean square error (RMSE)] are applied for model selection and validation. The estimates of AIC, AD*, KS test statistic and RMSE for the three competitive models are given in Table 3.

**Table 3:** Estimates of AIC, AD*, KS test statistic and RMSE for the models

Threefold mixture models	AIC	AD*	KS test	RMSE
Threefold Weibull	965.5942	0.6272	0.1068	0.0247
Weibull–Normal–Exponential	963.2532	0.5278	0.0876	0.0209
Normal–Lognormal–Weibull	964.6492	0.4781	0.0877	0.0217

From Table 3, we found that the Weibull–Normal–Exponential mixture model contains the smallest values of AIC and RMSE and the Normal–Lognormal–Weibull mixture model contains the smallest value of AD* test statistic among all of the mixture models. Hence, it can be concluded that, among these mixture models, Weibull–Normal–Exponential mixture model can be selected as the best model for hydraulic pump failure data according to the values of AIC and RMSE.

We have also applied the Kolmogrov–Smirnov (KS) test statistic as a goodness-of-fit test for these threefold mixture models. At the 5 % level of significance, with *n* = 102, the critical value of the Kolmogorov–Smirnov one-sample test is $1.36/\sqrt {102} = 0.135$ (Siegel and Castellan 1988). Since the observed value of the KS test statistic for all the threefold mixture models (given in Table 3) are less than the critical value, we cannot reject the null hypothesis, H_0_, that the observed data are from a population specified by these threefold mixture distribution. But we may consider that among all these three mixture models the Weibull–Normal–Exponential mixture model gives the smallest value for the KS test statistic.

According to Karim et al. (2015), let us introduce the following notations:

*q*: Probability that the pump is scrapped and replaced by a new one under service exchange
1 – *q*: Probability that the pump is not scrapped and reconditioned under service exchange
*p*: Probability that the item used in service exchange is installed correctly
1 – *p*: Probability that the item used in service exchange is not installed correctly
*F*_*N*_(*t*): Failure distribution of new item installed correctly
*F*_*R*_(*t*): Failure distribution of reconditioned item installed correctly
*F*_*I*_(*t*): Failure distribution of incorrectly installed item (new or reconditioned)

It is easily seen (using the conditional approach) that the time to failure of an item used in service exchange is given by a distribution function (Karim et al. 2015)

4 $$G_{3} \left( t \right) = \left( {1 - p} \right)F_{I} \left( t \right) + \left( {1 - q} \right)pF_{R} \left( t \right) + qpF_{N} \left( t \right)$$

Note that the MTTF (mean time to failure) for a new item installed correctly > MTTF for a reconditioned item installed correctly > MTTF for an item (new or reconditioned) installed incorrectly. If we select the Weibull (β, η)–Normal (μ, σ)–Exponential (δ) mixture model as the best model for the data, then according to the Table 4 of Karim et al. (2015), we can write

5 $$p_{3} = \left( {1 - p} \right),\quad p_{1} = \left( {1 - q} \right)p \quad {\text{and}} \quad p_{2} = qp$$

6 $$F_{3} \left( {t;\delta } \right) = F_{I} \left( t \right),\quad F_{1} \left( {t;\beta ,\eta } \right) = F_{R} \left( t \right) \quad {\text{and}}\quad F_{2} \left( {t;\mu ,\sigma } \right) = F_{N} \left( t \right)$$

**Table 4:** Optimal $T^{*}$ and $J\left( {T^{*} } \right)$ for different values of $\xi$

Model	Optimal values	Additional cost			
		$\xi$ = 70,000	$\xi$ = 90,000	$\xi$ = 110,000	$\xi$ = 130,000
Threefold Weibull	$T^{*}$	14,631	14,484	14,377	14,295
	$J\left( {T^{*} } \right)\;$	10.40593	11.43373	12.45314	13.46734
Weibull–Normal–Exponential	$T^{*}$	14,468	14,361	14,286	14,230
	$J\left( {T^{*} } \right)\;$	10.33359	11.33318	12.3265	13.31607
Normal–Lognormal–Weibull	$T^{*}$	14,476	14,368	14,291	14,234
	$J\left( {T^{*} } \right)\;$	10.32712	11.32669	12.31991	13.30929

Using the estimates of *p*_1_, *p*_2_ and *p*_3_ from Table 2 in Eq. (5), we get the estimates of *p* = 0.8326 and *q* = 0.6096.

## Optimum maintenance cost

Obtaining the solution to the problem involves building a model and deciding on the optimal age for PM action requires an objective function. The objective function is the asymptotic expected cost per unit time. Note that every time instant an exchanged pump is put into operation can be viewed as a renewal point for a renewal process characterizing the replacements of pumps over time. The time between two successive renewal points defines a cycle. The asymptotic expected cost per unit time can be obtained as the ratio of the expected cycle cost (ECC) and the expected cycle length (ECL).

The time to failure for a pump, *X*, is a random variable with distribution function *F*(*x*). A PM action results if $X \ge T$ in which case the cycle length is $T$ with probability *R*(*T*). A CM action results when $X < T$ and the cycle length is $X$. As a result ECL is given by

7 $$ECL = \int\limits_{0}^{t} {tf(t) + TR(T) = \int\limits_{0}^{T} {R(t)dt} }$$

Let $C_{f}$ and $C_{p}$ denote the average cost of a CM and a PM replacement respectively. We will discuss the derivation of this cost later in the section. As a result ECC is given by

8 $$ECC = C_{f} F(T) + C_{p} R(T)$$

From (7) and (8) we have the asymptotic average cost per unit time given by

9 $$J(T;F(.)) = \frac{{C_{f} F(T) + C_{p} R(T)}}{{\int\nolimits_{0}^{T} {R(t)dt} }}$$

$T^{*}$, the optimal *T*, is the value that yields a minimum for $J\left( {T;F\left( . \right)} \right)$.

The optimal $T$ depends on the average cost of each CM and PM. Like Karim et al. (2015), we use the following additional notations and assumptions.

$$C_{n}$$: Sale price for new pump ($80,000).

$$C_{r}$$: Cost (charged by the service agent) for reconditioning a pump under CM or PM action ($60,000).

$$\xi$$: Additional cost (due to downtime, loss in revenue, etc.) resulting from CM action. We look at values of $$\xi$$ = $70,000, $90,000, $110,000 and $130,000.

A maintenance action involves replacement by a new item or a reconditioned item with probabilities $q$ and $\left( {1 - q} \right)$ respectively. As a result, the average cost of a PM action is $C_{p} = qC_{n} + \left( {1 - q} \right)C_{r}$ and of a CM action is $C_{f} = C_{p} + \xi$. The optimal $T^{*}$ is obtained using (9) with threefold mixture cdf $F(t) = G_{3} (t)$ and the optimal expected cost per unit time is given by $J\left( {T^{*} ;F\left( . \right)} \right)$ i.e., $J(T^{ * } ;G_{3} ( \cdot ))$.

Here we can see that, the optimal $T^{*}$ depend on the additional cost $\xi$. The optimal $T^{*}$ and optimal expected cost per unit time $J\left( {T^{*} } \right)$ on various values of $\xi$ for the three different threefold mixture models has been estimated. These results are given in Table 4, from where it can be seen, for every model, the optimal $T^{*}$ decrease and optimal $J\left( {T^{*} } \right)$ increasing with $\xi$ increases, as to be expected.

Table 4 indicates that the threefold Weibull mixture model gives a bit larger optimal maintenance period $T^{*}$ than other two models, however the Weibull–Normal–Exponential model shows a reduction in the maintenance cost than the threefold Weibull mixture model for all $\xi$.

## Conclusion

Proper data management (data collection and analysis) is very important for effective maintenance of any engineered object. Data is critical for building and selecting suitable statistical models and model provides new insights for improvements to maintenance operations.

This paper has dealt with a real case study to illustrate how statistical models can be selected and applied for estimating optimum maintenance period and cost of a hydraulic pump. It is recommended that the Weibull–Normal–Exponential mixture model can be selected as the best model for hydraulic pump failure data among three competitive models. This model suggests the optimum maintenance period for the pump that reduces the maintenance cost. Annotated R code is provided for analyzing hydraulic pump failure data with Weibull–Normal–Exponential mixture model. The code can be modified easily to apply other threefold mixture models.

## Appendix

R codes for analyzing pump failure data with Weibull–Normal–Exponential mixture model.
